# Supplementary figures and images for: Establishment of an orthodontic retention mouse model and the effect of anti-c-Fms antibody on orthodontic relapse
Source: PLoS One. 2019 Jun 19;14(6):e0214260. doi: 10.1371/journal.pone.0214260 (PMC6583981; doi:10.1371/journal.pone.0214260)

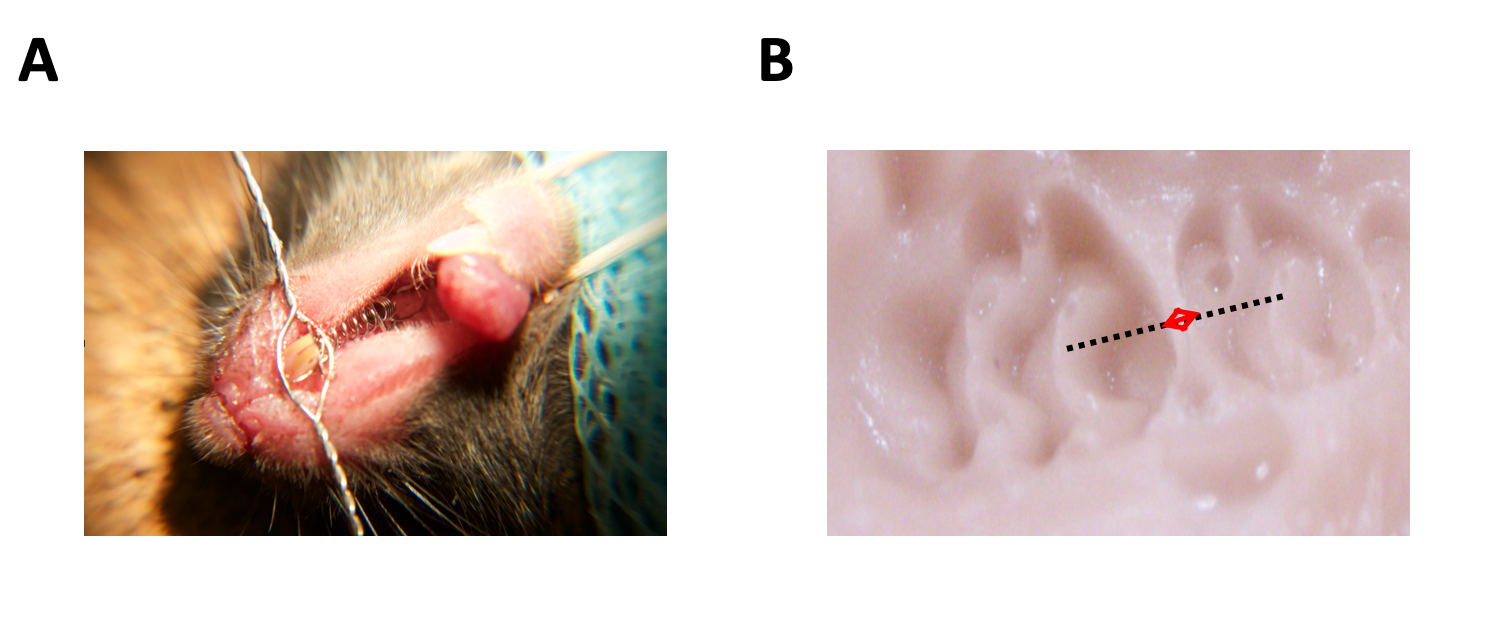

Supplement: S1 Fig — (A) A nickel-titanium closed-coil spring is fixed between the upper incisors and the upper-left first molar. A 0.1-mm stainless steel wire is used to move the first molar in a mesial direction. (B) Photograph of the silicone impression after tooth movement. The dashed line connecting the central fossae of the first and second molars was used to measure the distance of tooth movement (from the distal marginal ridge of M1 to the mesial marginal ridge of M2) (red double arrow). (TIF) [file pone.0214260.s001.tif]

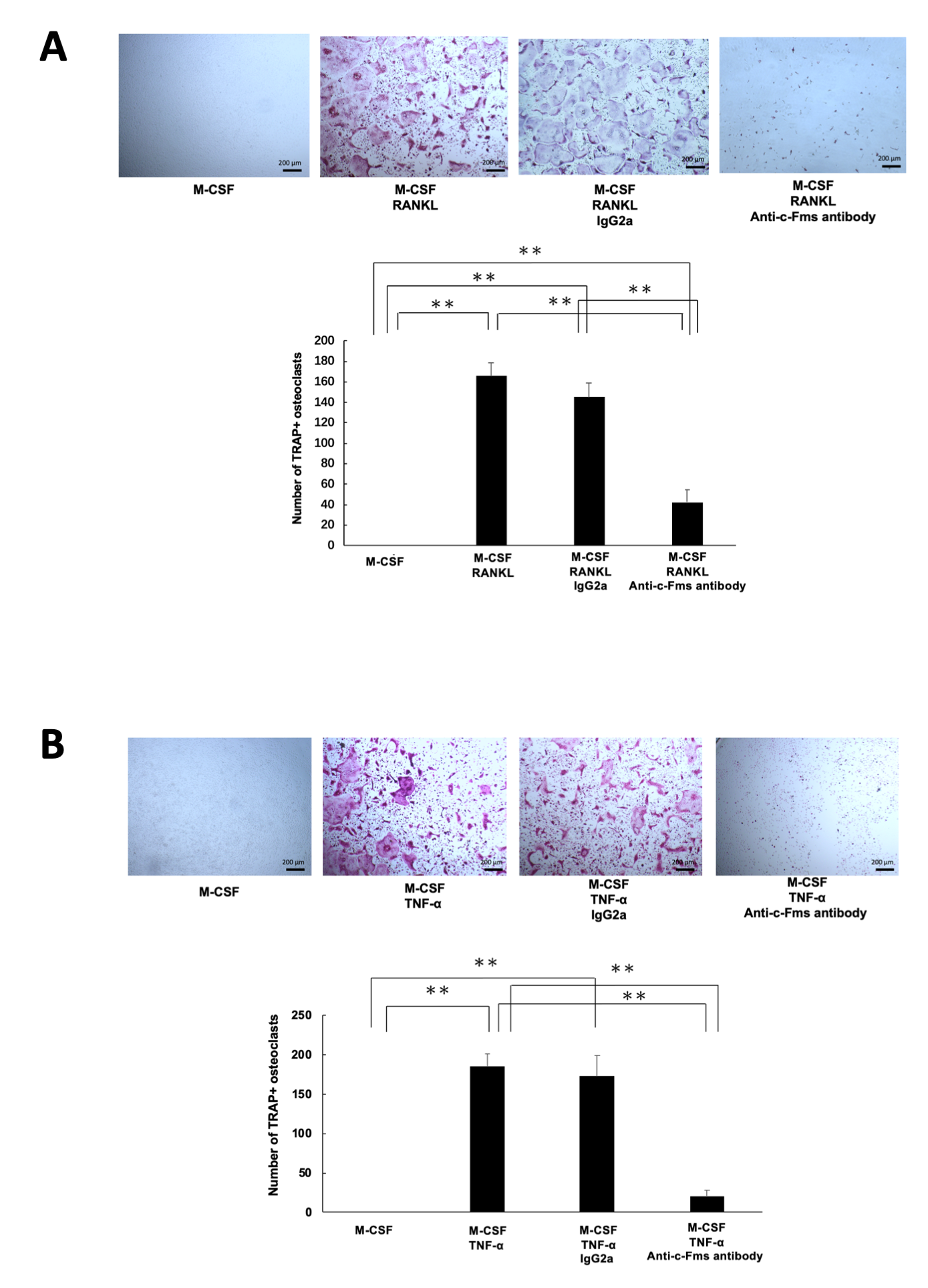

Supplement: S2 Fig — (A)Microscopic images and numbers of TRAP-positive cells. Osteoclast precursors were treated with macrophage colony-stimulating factor (M-CSF) alone, M-CSF with RANKL, M-CSF with RANKL and IgG2a (isotype control), and M-CSF with RANKL and anti-c-Fms antibody for 5 days. Cells were fixed and stained with TRAP staining. Results were expressed as mean ± S.D. Statistical differences were detected by using Scheffe’s tests (n = 4; **p < 0.01). Scale bars = 200 μm. (B) Microscopic images and numbers of TRAP-positive cells. Osteoclast precursors were treated with macrophage colony-stimulating factor (M-CSF) alone, M-CSF with TNF-α, M-CSF with TNF-α and IgG2a (isotype control), and M-CSF with TNF-α and anti-c-Fms antibody for 5 days. Cells were fixed and stained with TRAP staining. Results were expressed as mean ± S.D. Statistical differences were detected by using Scheffe’s tests (n = 4; **p < 0.01). Scale bars = 200 μm. (TIF) [file pone.0214260.s002.tif]

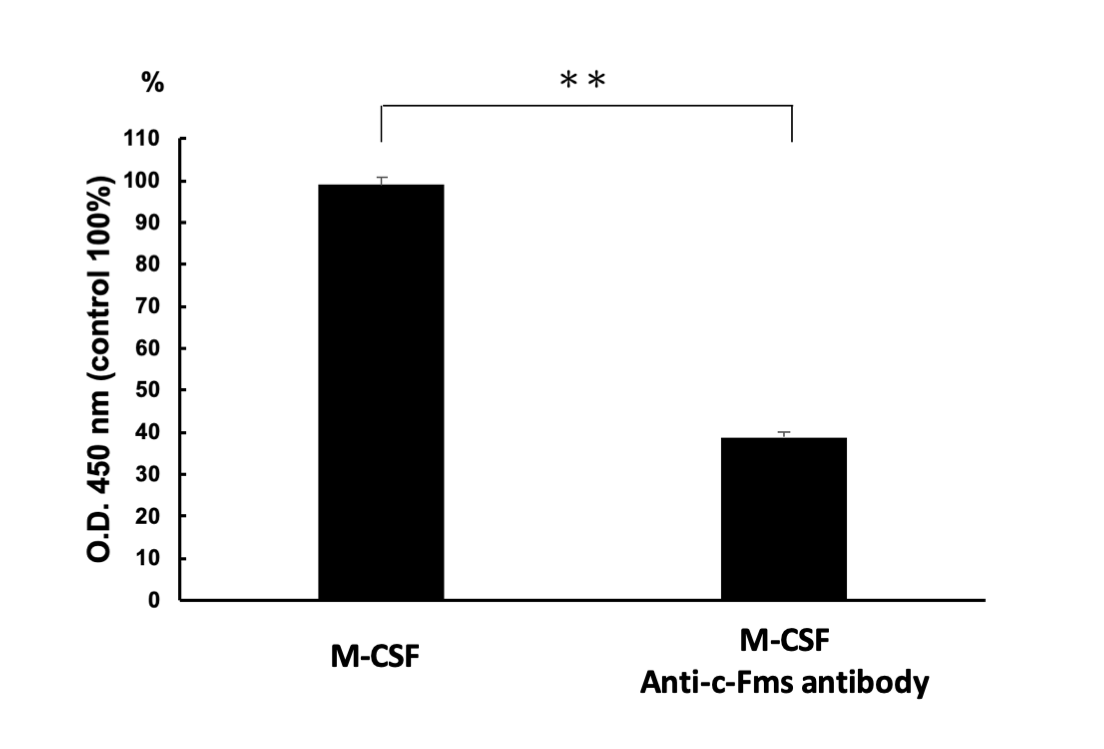

Supplement: S3 Fig — Cell viability of osteoclast precursor cells treated with M-CSF and with or without anti-c-Fms antibody (1,000 ng/mL) for 3 days. Cell counting kit-8 was used to measure viability. Data are presented as a percentage to compare the relative activity of the wells containing M-CSF+anti-c-fms versus the wells containing M-CSF alone and expressed as mean ± S.D. of four cultures. Statistical differences were detected by using Student’s t-tests (n = 4; **p < 0.01). (TIF) [file pone.0214260.s003.tif]
