# Supplementary material for: Establishment of an orthodontic retention mouse model and the effect of anti-c-Fms antibody on orthodontic relapse
Source: PLoS One. 2019 Jun 19;14(6):e0214260. doi: 10.1371/journal.pone.0214260 (PMC6583981; doi:10.1371/journal.pone.0214260)
Supplement: S1 Text — (DOCX) [file pone.0214260.s004.docx]

**Material and Methods**

**Cell culture experiments**

The bone marrow cells from femoral and tibial epiphyses from 8–10-week-old male C57BL/6J mice was harvested and then cultured in α-MEM containing 10% FBS, 100 IU/mL penicillin G (Meiji Seika, Tokyo, Japan) and 100 μg/mL streptomycin (Meiji Seika) with M-CSF. Adherent cells were harvested using trypsin-EDTA solution (Sigma-Aldrich). Harvested cells were then cultured in the presence of M-CSF for 3 days. Cells were recognized as osteoclast precursors. Osteoclast precursors were seeded at 5 × 10^4^ cells per 200 μL of culture medium in a 96-well plate and cultured for 5 days in medium containing M-CSF (100 ng/mL); M-CSF (100 ng/mL) and RANKL (100 ng/mL) or TNF-α (100 ng/mL); M-CSF (100 ng/mL), RANKL (100 ng/mL) or TNF-α (100 ng/mL), and anti-c-Fms antibody (100 ng/mL) or IgG2a control antibody (100 ng/mL). After fixation with 4% PBS-buffered formaldehyde, cells were permeabilized with 0.2% Triton X-100 and TRAP staining was performed to visualize active osteoclasts. TRAP-positive cells containing three or more nuclei were counted under a light microscope.

**Cell Viability Assay for Osteoclast Precursors.**

Osteoclast precursors were seeded in a 96-well plate (1 × 10^4^ cells in 200 μl medium per well) and incubated with M-CSF (100 ng/ml) with or without anti-c-Fms antibody (1000 ng/ml). After 3 days of incubation, the cells were washed with PBS and cultured in 100 μl culture medium of each well. 10 μl cell counting kit-8 (Dojin, Kumamoto, Japan) solution was added to each well, and the plate was further incubated for 2 h at 37°C. Absorbance at 450nm was measured by a microplate reader for each well.
